# Archaeal Hel308 suppresses recombination through a catalytic switch that controls DNA annealing

**DOI:** 10.1093/nar/gkad572

**Published:** 2023-07-06

**Authors:** Rebecca J Lever, Emily Simmons, Rebecca Gamble-Milner, Ryan J Buckley, Catherine Harrison, Ashley J Parkes, Laura Mitchell, Jacob A Gausden, Sanja Škulj, Branimir Bertoša, Edward L Bolt, Thorsten Allers

**Affiliations:** School of Life Sciences, University of Nottingham, Nottingham NG7 2UH, UK; School of Life Sciences, University of Nottingham, Nottingham NG7 2UH, UK; School of Life Sciences, University of Nottingham, Nottingham NG7 2UH, UK; School of Life Sciences, University of Nottingham, Nottingham NG7 2UH, UK; School of Life Sciences, University of Nottingham, Nottingham NG7 2UH, UK; School of Life Sciences, University of Nottingham, Nottingham NG7 2UH, UK; School of Life Sciences, University of Nottingham, Nottingham NG7 2UH, UK; School of Life Sciences, University of Nottingham, Nottingham NG7 2UH, UK; Department of Chemistry, Faculty of Science, University of Zagreb, Horvatovac 102a, HR-10000 Zagreb, Croatia; Department of Chemistry, Faculty of Science, University of Zagreb, Horvatovac 102a, HR-10000 Zagreb, Croatia; School of Life Sciences, University of Nottingham, Nottingham NG7 2UH, UK; School of Life Sciences, University of Nottingham, Nottingham NG7 2UH, UK

## Abstract

Hel308 helicases promote genome stability in archaea and are conserved in metazoans, where they are known as HELQ. Their helicase mechanism is well characterised, but it is unclear how they specifically contribute to genome stability in archaea. We show here that a highly conserved motif of Hel308/HELQ helicases (motif IVa, F/YHHAGL) modulates both DNA unwinding and a newly identified strand annealing function of archaeal Hel308. A single amino acid substitution in motif IVa results in hyper-active DNA helicase and annealase activities of purified Hel308 *in vitro*. All-atom molecular dynamics simulations using Hel308 crystal structures provided a molecular basis for these differences between mutant and wild type Hel308. In archaeal cells, the same mutation results in 160000-fold increased recombination, exclusively as gene conversion (non-crossover) events. However, crossover recombination is unaffected by the motif IVa mutation, as is cell viability or DNA damage sensitivity. By contrast, cells lacking Hel308 show impaired growth, increased sensitivity to DNA cross-linking agents, and only moderately increased recombination. Our data reveal that archaeal Hel308 suppresses recombination and promotes DNA repair, and that motif IVa in the RecA2 domain acts as a catalytic switch to modulate the separable recombination and repair activities of Hel308.

## Graphical Abstract

**Graphical Abstract:**
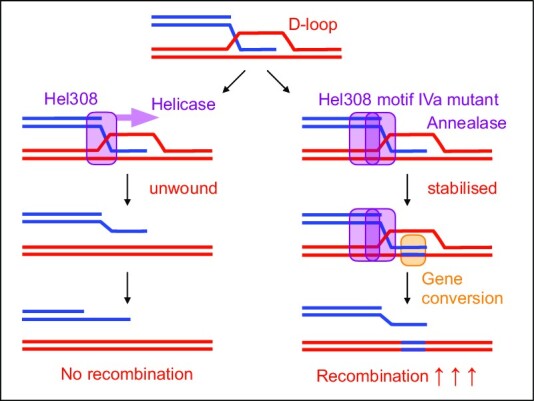

## INTRODUCTION

DNA helicases are required for homologous recombination (HR), which repairs DNA double-strand breaks by processes conserved across bacteria, archaea, and eukaryotes, reviewed recently (1,2). The key step of HR is formation of a displacement loop (D-loop) by a RecA-family recombinase, which catalyses the invasion of an unbroken duplex by the broken DNA strand, priming new DNA synthesis from the available 3′OH end. DNA synthesis in this context is eventually halted and the chromosome reconstituted by annealing and ligation of nascent DNA – a non-crossover outcome to recombination – or by resolution of branched DNA intermediates to produce a crossover outcome. In bacteria and eukaryotes, helicase enzymes modulate HR by antagonising or promoting D-loop formation (3–5). However, the factors that control the genetic outcomes of HR in archaea are unclear, including whether crossovers or non-crossovers predominate.

Hel308 helicases promote genome stability, they were identified by mutations in *Drosophila* and mouse that cause sensitivity to mitomycin C (MMC), nitrogen mustard and cisplatin (6–8). Archaeal Hel308, also called Hjm, was discovered by sequence homology with the human Hel308 protein, which is now called HELQ, and from similar biochemical characteristics of Hel308 and HELQ proteins *in vitro* (9–11). Hel308 translocates DNA with 3′ to 5′ directionality and is most effective at separating DNA strands within branched DNA structures, such as forked DNA and D-loops (9). DNA translocation by Hel308 is applied in nanopore sequencing of DNA and polypeptides (12,13).

Hel308 and HELQ interact with the single-strand DNA (ssDNA) binding protein RPA (14–16), providing Hel308 and HELQ with access to ssDNA that is required to trigger their ATP-dependent DNA translocation (9,17) and DNA annealing activities (16). Archaeal Hel308 also physically interacts with Holliday junction processing proteins PINA and Hjc (18,19). Thus, the biochemical properties and interactions of Hel308 implicate it in processes that rescue cells from DNA damage during replication; however, it is unclear exactly how these activities of Hel308 contribute to genome stability.

**Figure 1.**
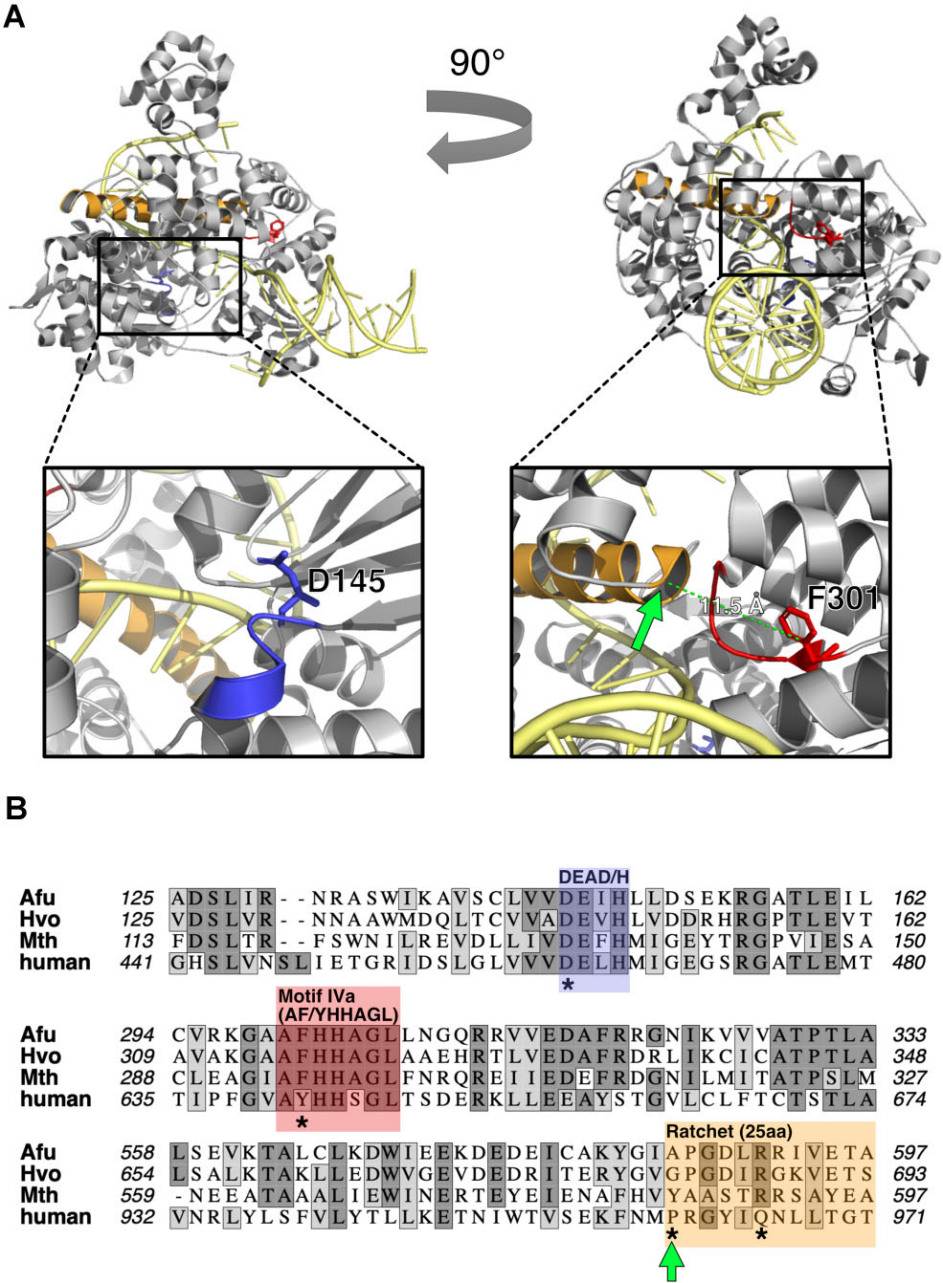
Hel308 motif IVa—a highly conserved motif of unknown function that abuts the single-strand DNA translocating ‘ratchet’ domain. (**A**) Atomic resolution structures for Hel308 from the archaeon *Archaeoglobus fulgidus* (*Afu*-Hel308) (PDB 2P6R) (20). Shown in orange is the conserved alpha helical ‘ratchet’ that is essential for Hel308 translocation along ssDNA (yellow) and shown in red are the residues of motif IVa. Green arrow indicates the residue (an alanine shown here in *Afu*-Hel308) located at the end of the ‘ratchet’ that contacts motif IVa. The inset panels detail (on left) the ATPase active site showing the critical aspartic acid residue in blue, and (on right) the proximity of the ‘ratchet’ (orange) to motif IVa (red). (**B**) Sequence alignment for *Afu*-Hel308, Hel308 from *Haloferax volcanii* (Hvo) and *Methanothermobacter thermautotrophicus* (Mth), and for human HELQ, the closest sequence homologue of Hel308. Residues highlighted with asterisks were mutated in this work for biochemical analysis (*Mth-*Hel308) and genetics (*Hvo-*Hel308).

Atomic resolution structures of archaeal Hel308 have provided detailed understanding of its ssDNA translocation and DNA unwinding mechanisms (20–22). In particular, a Hel308-DNA co-structure shows extensive molecular contacts between Hel308 and ssDNA, including at the ssDNA/dsDNA branchpoint of the substrate, which may explain its specificity for removing a lagging-strand from a model forked DNA *in vitro* (20). Hel308 translocates ssDNA 3′ to 5′ stepwise, using a ‘ratchet’ helix assisted by a DNA binding winged helix domain, in a mechanism that appears to be physically coupled to conformational movements directed by the RecA-like ATPase domains *via* a ‘linker’ region (20,23,24). Hel308 structures also highlight that within the RecA2 domain there is a motif termed IVa (AF/YHHAGL), which is conserved throughout Hel308, Ski2 and RecQ sub-families of Superfamily 2 helicases but is of unknown function (9) (Figure 1).

In this work, we investigated the biochemical function of motif IVa, using purified Hel308 from the euryarchaeon *Methanothermobacter thermautotrophicus*. This was complemented by a genetic analysis of Hel308 motif IVa, to correlate with its cellular function in the euryarchaeon *Haloferax volcanii*. By focussing on a conserved phenylalanine in motif IVa, and the contacts this residue makes within the atomic resolution structures, we generated mutant *M. thermautotrophicus* proteins that were hyperactive at DNA binding and DNA annealing. The corresponding *H. volcanii* mutants displayed increased non-crossover recombination by several orders of magnitude—but without any impact on cell growth or the repair of interstrand DNA crosslinks (ICLs). These separation-of-function mutations in motif IVa reveal that Hel308 has two distinct roles: the repair of DNA damage, and the suppression of HR.

## MATERIALS AND METHODS

*Mth-*Hel308 wild type and mutant proteins were purified with N-terminal (His)_6_ tags after over-expression in *Escherichia coli* BL21 A.I. cells (Invitrogen), as described in (9). Cells were harvested and resuspended in resuspension buffer (20 mM Tris–HCl, pH 8.0, 10% glycerol, 500 mM sodium chloride and 20 mM imidazole) for flash freezing in dry ice. Thawed cells were lysed by sonication and clarified to obtain soluble cell proteins that were loaded into a Ni-NTA column with Hel308 proteins eluting in a linear gradient (20–500 mM) of imidazole. Fractions containing Hel308 were revealed using SDS PAGE in 8% (w/v) acrylamide/bis-acrylamide (37.5:1) gels, for pooling and dialysis into buffer A (20 mM Tris–HCl, pH 8.0, 10% glycerol, 100 mM potassium acetate, 2 mM DTT). This was loaded in to a HiTrap heparin column with Hel308 eluting approximately mid-way in a linear gradient of 100 mM to 2 M potassium acetate, peak fractions evaluated from SDS PAGE in 8% (w/v) acrylamide/bis-acrylamide (37.5:1) gels. Hel308 was dialysed into buffer A containing 30% glycerol, flash frozen and stored at -80°C. Concentrations of purified proteins (mg/ml) were measured against a BSA standard curve in the Bradford assay, and by absorbance measurement at 280 nm in a Denovix DS11 nanodrop (Cambridge BioScience), to generate a mean protein concentration from the two measurement methods.

*In vitro* analysis of Hel308 proteins used DNA substrates shown in Supplementary Table S5. Electrophoretic Mobility Shift Assays (EMSAs) monitored stable binding of Cy5 labelled DNA with protein. Reaction mixtures contained 20 mM Tris–HCl pH 7.5, 100 μg/ml BSA, 7% (v/v) glycerol, 25 mM DTT, 5 mM EDTA and 25 nM fluorescently labelled DNA and protein as stated in Figures. For EMSAs, DNA binding by proteins was quantified in ImageJ from TIFF images, acquired by Typhoon scanning of the Cy5 signal of gels, using unbound DNA control lanes for each set of reactions to represent zero DNA protein–DNA complex. Values of DNA binding obtained against unbound DNA were plotted in Prism using non-linear regression analysis to obtain *K*_d_ and *R*^2^ values. Reactions of Hel308 with duplex DNA were incubated at 37°C for 10 min prior to addition of Orange G loading dye and separation on 5% (w/v) acrylamide/bis-acrylamide (37.5:1) TBE gels. Gels were imaged and analysed as for the EMSAs, with the ‘no-protein’ control signifying zero helicase activity for each set of reactions. FRET analyses of DNA helicase and single strand DNA annealing activities of Hel308 proteins were monitored using Cy5 and Cy3 labelled DNA substrates as described in (25).

### Genetic manipulation of *H*.*volcanii*

Growth and transformation of *H. volcanii*, isolation of genomic DNA, and verification of *hel308* deletion and gene replacement mutants by Southern blotting was carried out as described previously (26). *H. volcanii* strains, plasmids and primers are listed in Supplementary Tables S1, S3 and S4, respectively. *E. coli* strains are listed in Supplementary Table S2.

To generate the *hel308* deletion plasmids, upstream and downstream flanking regions of *hel308* were PCR amplified from a chromosomal DNA fragment in pTA415 containing *hel308*, using primer pairs HQEPF/hel308Nde5R and cgiNde5F/HQEPR, respectively. PCR fragments were ligated together via the internal NdeI site and cloned in the HindIII and XbaI sites of pTA131 (26) to generate the *hel308* deletion plasmid, pTA1254; in pTA1254 the essential downstream *cgi* gene is not disrupted (Figure 4). pTA1273 is derivative of pTA1254 with a *trpA+* marker at the *hel308* locus.

The *hel308-D145N* mutation was introduced by overlap extension PCR amplification of *hel308* using primers PBSF, HQD145NR, HQD145NF and ski2R. An 806 bp BsrG1-NsiI fragment of the *hel308-D145N* product was cloned in the BsrGI and NsiI sites of the *hel308* plasmid pTA1316 to generate the *hel308-D145N* gene replacement construct, pTA1335. The *hel308-F316A* mutation was introduced using primers Hel308FInt, Hel308F316AR, Hel308F316AF and Hel308EcoR, the 977 bp BspEI-EcoRI fragment of the *hel308-F316A* product was cloned in the EcoRI and BspEI sites of p1316 to generate the *hel308-F316A* gene replacement construct, pTA1642. The *hel308-D145N-F316A* gene replacement construct pTA1952 was generated using the same overlap extension PCR strategy as *hel308-F316A*, but using the *hel308-D145N* gene replacement construct pTA1335 as the template. All plasmid constructs confirmed by DNA sequencing. The *hel308* point-mutant allelic replacement plasmids pTA1335, pTA1642 and pTA1952 were used to replace the *trpA +* marked *hel308* deletion allele, generating H2400, H2397 and H3926, respectively. 5-FOA-resistant colonies were screened via colony hybridization using an internal *hel308* radio-labelled probe (ski2F/ski2R PCR product). Approximately 50% contained the *hel308*-mutant allele and were further verified via Southern blot (note that PCR verification of *H. volcanii* strains is unreliable due to the highly polyploid nature of the genome).

*H. volcanii* growth assays in Hv-YPC broth were determined using an Epoch 2 Microplate Spectrophotometer (BioTek) as described previously (27) with these modifications: growth of 150 μl cultures in 96-well microtiter plates at 45°C with double orbital shaking at 807 rpm was performed for 72h. The optical density at 600 nm was measured every 15 min and the generation time was calculated between OD_600_ values of 0.08–0.16 using the formula:


\begin{equation*}\begin{array}{@{}*{1}{l}@{}} {G = \frac{t}{n}}\\ {n = \frac{{{\mathrm{log}}b - {\mathrm{log}}B}}{{{\mathrm{log}}2}}} \end{array}\,\,\,\,\,\,\,\,\,\,\,\,\,\,\,\,\,\,\,\,\,\,\,\,\,\,\,\,\,\,\,\,\,\,\begin{array}{@{}*{1}{l}@{}} {{\mathrm{G}} = {\mathrm{generation time}}}\\ {{\mathrm{t}} = {\mathrm{time}}}\\ {{\mathrm{n}} = {\mathrm{number of generations}}}\\ {{\mathrm{b}} = {\mathrm{end }}{{\mathrm{A}}}_{600}}\\ {{\mathrm{B}} = {\mathrm{start }}{{\mathrm{A}}}_{600}} \end{array}\end{equation*}

Episomal complementation of Δ*hel308* in *H. volcanii* was determined by growth assays as above, with these modifications: cultures were grown in Hv-YPC broth with excess uracil (0.05 mg/ml), thymidine (0.048 mg/ml), and hypoxanthine (0.048 mg/ml).

DNA damage assays for sensitivity to mitomycin C (0–0.02 μg/ml) were carried out as described in (28), and recombination assays (*H. volcanii* plasmid x chromosome) were carried out as described in (27,28), and are illustrated in Figure 5A.

Molecular dynamics analysis of Hel308 used as the starting structure the *M. thermautotrophicus* wild type Hel308 protein obtained through homology modelling to the crystal structure of *A. fulgidus* Hel308 (PDB code 2P6R, resolution 3.00 Å) (20), with matching tertiary structure obtained in Blast protein comparative structure modelling from UniProt Protein Knowledgebase Homology (29,30). Amino acid sequence identity between Mth and Afu Hel308 2P6R is 34%. Models were constructed using MODELLER 9 (31) and Chimera 1.13.1 (32). In order to study structural and dynamical properties of the *M. thermautotrophicus* Hel308 protein, four different systems were prepared and subjected to molecular dynamics (MD) simulations: (1) Hel308 without DNA, (2) Hel308 in complex with DNA, (3) F295A mutant without DNA and (4) F295A mutant in complex with DNA. All systems were prepared for simulations using the CHARMM-GUI Input Generator (33,34). Side chains of all arginine and lysine residues were positively charged, while side chains of glutamate and aspartate residues were negatively charged. Protein and DNA atoms were parametrized using CHARMM36m force field (35). Explicit solvent model of TIP3P model of water molecule was used. Systems with/without DNA contained approximately 90 000/56 800 water molecules, and 55/17 potassium ions necessary to neutralize the net charge of system.

Prior to MD simulations, each system was energy minimized (geometry optimized) using 5000 steps of steepest descent (SD) algorithm. Equilibration phase lasted 125 ps using 1 fs time step and the position restraint of protein backbone atoms with harmonic potential of 400 kJ mol^−1^ nm^−2^. Production phase lasted for 1.0 μs with a time step of 2 fs. Lincs algorithm was applied and periodic boundary conditions were used. Size of a periodic rectangular box was approximately 14 nm × 14 nm × 14 nm for systems consisting of Mth Hel308–DNA complexes and 12 nm × 12 nm × 12 nm for systems of Mth Hel308 without DNA. Isobaric-isothermal ensemble (NPT) at T = 310 K was used for the production phase. Temperature was maintained via Nosé−Hoover thermostat (36,37) with a coupling constant of 1.0 ps^−1^. Semi-isotropic Parrinello − Rahman barostat (38) was used to regulate pressure of 1.013 bar and controlled with a time constant for pressure coupling of 5 ps^−1^. Long range electrostatic interactions were calculated by the particle-mesh Ewald (PME) method with real space Coulomb interactions cut-off at 1.2 nm using a Fourier spacing of 0.12 nm and Verlet cut-off scheme. All simulations were conducted using GROMACS 2018.6 software package (39) and visualized with VMD (Visualise Molecular Dynamics) (40).

## RESULTS

### Mutation of Hel308 motif IVa causes hyper-active DNA binding, unwinding and annealing

**Figure 2.**
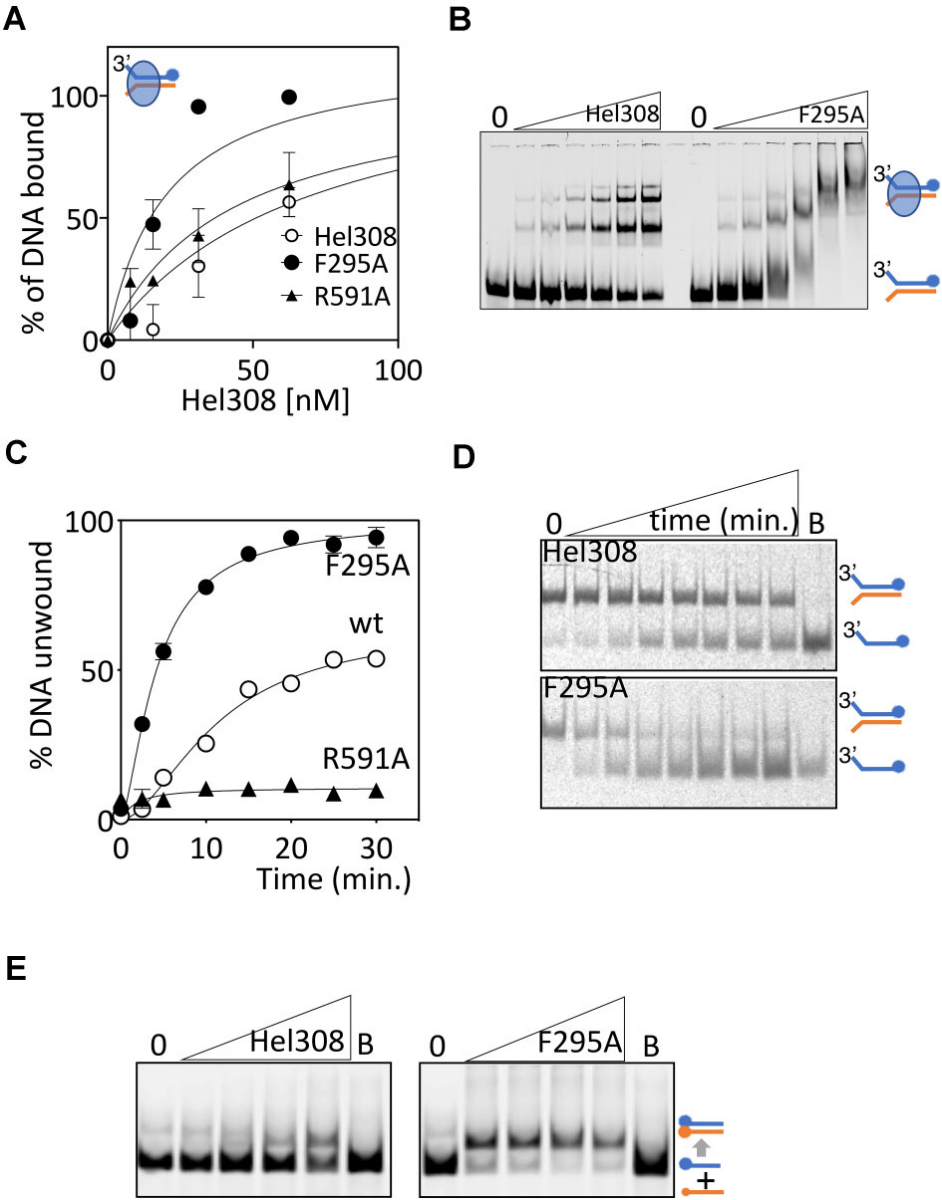
Hel308 motif IVa mutant F295A is hyperactive at DNA binding and is predicted to alter conformational movement of Hel308. (**A**) EMSAs of Hel308 proteins binding to a forked DNA substrate (25 nM) end labelled with Cy5. Hel308 proteins were used at 7.5, 15.0, 30 and 62.5 nM for the data points shown, which are mean values from *n* = 3 showing standard error of the mean. (**B**) Summarises EMSAs quantified with a representative gel showing Hel308 reactions at 7.5, 15.0, 30, 62.5, 125 and 250 nM. (**C**). Data is helicase unwinding by Hel308 proteins (80 nM) of a forked duplex DNA (25 nM) end labelled with Cy5 on one strand as indicated, in end point assays for the given time. Hel308^F295A^ was hyperactive compared with wild type (wt) Hel308 and Hel308^R591A^ that is mutated in the DNA strand separating arginine residue (20). The data points are means from *n* = 2, with error bars for standard error of the mean. (**D**) Shows a representative gel from the helicase assays quantified in (**C**), with B denoting a boiled reaction to dissociate all DNA duplex. Time points taken: 0, 2.5, 5, 10, 15, 20, 25 and 30 min. (**E**) Shows summary gels of DNA annealing (15 nM) by the Hel308 proteins as indicated (50, 100, 200 and 400 nM).

Hel308-DNA interactions have been detailed at atomic resolution from *Archaeoglobus fulgidus* (*Afu*-Hel308 PDB 2P6R) (20) but their contributions to DNA repair and recombination remain unclear. To address this, we used *Afu-*Hel308 structures to identify conserved regions of unknown function, and to mutate them to investigate their role *in vitro* and *in vivo*. The highly conserved Hel308 RecA2 domain region termed ‘motif IVa’ (AF/YHHAGL) is located intriguingly close to the α-helical ‘ratchet’ domain 4 that is essential for DNA translocation (Figure 1 and Supplementary Movie 1). We purified mutant Hel308 proteins from the euryarchaeon *Methanothermobacter thermautotrophicus* (*Mth*-Hel308) with amino acid substitutions in either motif IVa (Hel308^F295A^) or the ratchet (Hel308^R591A^) (Supplementary Figure S1A). All Hel308 proteins formed stable complex with forked DNA in electrophoretic mobility shift assay (EMSA) gels, although Hel308^F295A^ gave significantly increased protein-DNA binding stability when compared with the other Hel308 proteins: *K*_d_ 19.3 nM (*R*^2^ = 0.847) compared with *K*_d_ 68.25 nM (*R*^2^ = 0.890) for wild type Hel308 (Figure 2A), calculated as described in Methods. This was clearly apparent in a distinct pattern of DNA-Hel308^F295A^ complexes that formed more readily than wild type Hel308 (Figure 2B), manifested as full binding of all DNA substrate as DNA-Hel308^F295A^, compared with availability of free (unbound) DNA when wild type Hel308 was used at the same concentration.

Increased affinity for DNA by Hel308^F295A^ corresponded with increased ATP-dependent DNA unwinding of the same forked DNA substrate by Hel308^F295A^, as measured in end point assays as a function of time, compared with wild type Hel308 and Hel308^R591A^ (Figure 2C, D, and Supplementary Figure S4). We further tested whether Hel308 can anneal complementary single stranded DNA independently of ATP, a recently discovered activity of human HELQ (16,25). Hel308 annealed complementary 70 nt DNA strands, observed in gels with increasing Hel308 concentration, an activity that was robustly stimulated when replacing wild type Hel308 with Hel308^F295A^ (Figure 2E). Therefore, Hel308^F295A^ protein is hyperactive in all forms of DNA processing by Hel308.

### Molecular dynamics and *in vitro* analyses identify separable control of DNA unwinding and DNA annealing

**Figure 3.**
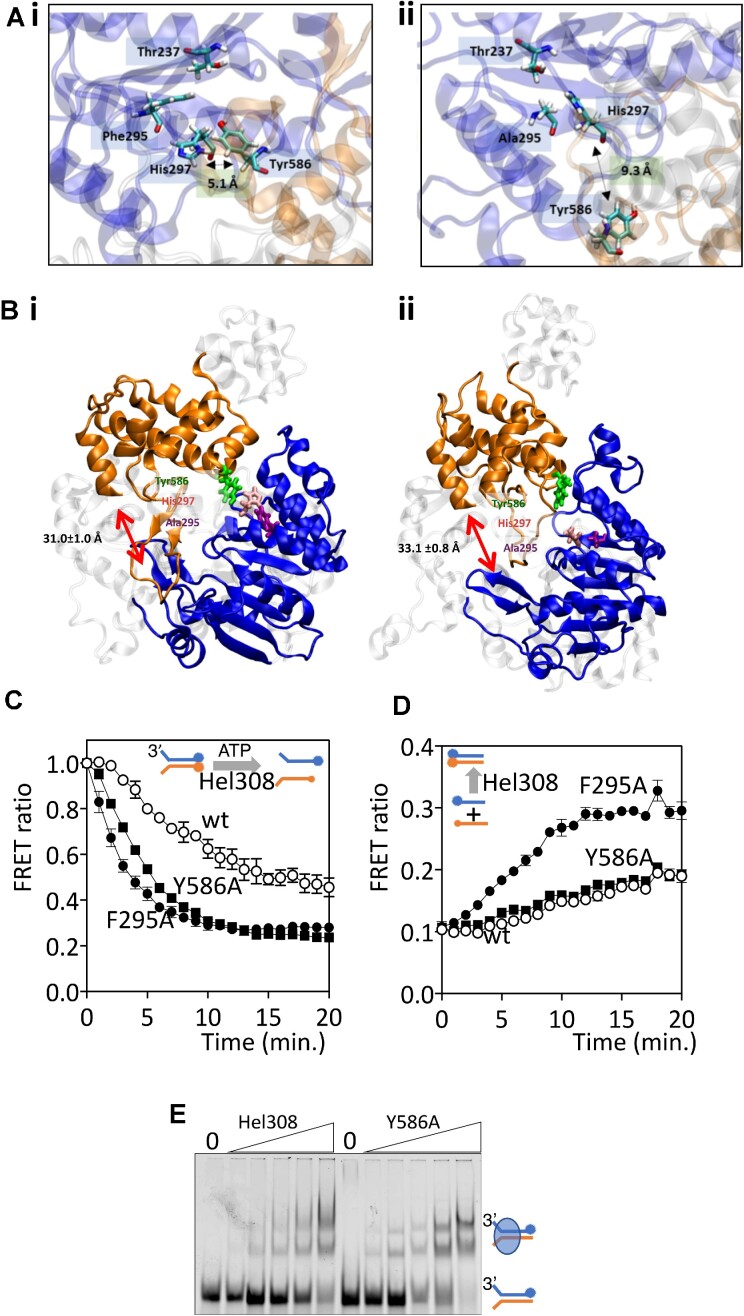
Hel308 motif IVa mutant F295A is hyperactive at DNA helicase and DNA annealing activities. (**A**) Molecular dynamics simulations using the Mth Hel308 structure were obtained by homology modelling in PHYRE2, using the Afu Hel308-DNA crystal structure as a template. Panel (i) shows positioning of motif IVa (domain 2, blue) Phe295 and His297 relative to ‘ratchet’ domain 4 (orange) Tyr586, which is substantially altered in the modelling, (ii) by mutation of Phe295 to alanine. (**B**) Hel308^F295A^ alters the relative topology of domain 2 (blue) and domain 4 (orange) in Mth Hel308, moving the domains away from one another resulting in a more ‘open’ architecture. The average value and the standard deviation of the distance between the centre of masses of domain 2 (residues 194–403) and domain 4 (residues 498–634) were calculated during the simulations are shown and labelled by arrows. (**C**) Real-time FRET measurements of DNA unwinding (15 nM) by Hel308 proteins (80 nM) as indicated. The forked DNA substrate had an additional 3′ Cy3 end label to detect loss of FRET as DNA is unwound, shown as a lower FRET ratio that is calculated from no-protein controls (see methods). The plots are means from *n* = 3 and show bars for standard error of the mean. (**D**) Real-time FRET measurement of single strand DNA annealing by Hel308 proteins (200 nM). Complementary 70nt DNA strands (50 nM of each) were end labelled with 5′ Cy5 or 3′ Cy3 to detect annealing by increased FRET, shown as higher FRET ratios calculated from no-protein control reactions (see methods). (**E**) EMSA summarising that Hel308^Y586A^ binds to DNA similarly to wild type Hel308. Proteins were each at 20, 40, 80, 160 and 320 nM mixed with forked DNA (25 nM).

To gain more insight into the role of Hel308 motif IVa, and why this is modified by mutation of Phe-295, we deployed molecular dynamics (MD) simulations, comparing conformations of Hel308 proteins modelled unbound or bound to DNA. We first used the experimentally determined *Afu-*Hel308 structure in comparison with *Afu-*Hel308^F301A^, equivalent to *Mth-*Hel308^F295A^. In our initial tests, visualisation and comparison of root mean square deviation (RMSD) values for *Afu-*Hel308^F301A^ with wild type *Afu-*Hel308 indicated that domain 2 is in a rigidly ’open’ conformation in the mutant compared with wild type (Supplementary Figure S2), and increased root mean square fluctuations (RMSFs) for residue side chains indicated increased positional flexibility in *Afu-*Hel308^F301A^ compared with wild type *Afu-*Hel308 resulting from lost hydrophobic interactions (Supplementary Figure S2). We then applied these observations to a modelled structure of *Mth-*Hel308 (Figure 3A), revealing the likelihood of specific π-π interactions between the side chains of Phe-295 and His-297 (both in domain 2), as well as between His-297 (domain 2) and Tyr-586 of the essential helicase ‘ratchet’ domain 4 (Figure 3A and Supplementary Figure S4A). In *Mth-*Hel308^F295A^ the specific distance between Ala-295 (mutant) and Tyr-586 residues was nearly doubled, compared with the wild-type Phe-295 to Tyr-586 distance (Figure 3Ai and ii). This was accompanied by a significant separation between domains 2 and 4, measured as a predicted increase in mean distance between their centres of masses from 31.0 ± 0.1 Å in the wild-type protein, to 33.1 ± 0.08 Å in Hel308^F295A^, which was observable in multiple orientations of the proteins (Table 1, Figure 3Bi and ii, and Supplementary Figure S3E and S3F). The same changes in protein structure were not apparent during simulations of protein-DNA complexes, due to the presence of DNA increasing the rigidity of protein structures in these analyses.

**Table 1.** *M. thermautotrophicus* Hel308 motif IVa mutation alters molecular dynamics of Hel308. Wild-type Hel308 and Hel308^F295A^ were compared for mean distances between amino acids in the non-covalent interaction network Thr237-Phe295-His297-Tyr567, and between the centre of masses for domains 2 and 4 (D2–D4)

**WT Mth Hel308—mean distance and standard deviation (Å)**			
Thr237-Phe295	Phe295-His297	His297-Tyr586	D2–D4
5.9 ± 0.4	7.1 ± 1.2	5.1 ± 1.6	31.0 ± 1.0
**Mutant F295A—mean distance and standard deviation (Å)**			
Thr237-Ala295	Ala295-His297	His297-Tyr586	D2–D4
4.6 ± 0.6	5.4 ± 1.1	9.3 ± 3.5	33.1 ± 0.8

Our molecular dynamics analyses therefore identified significant inter-domain interactions in Hel308 between Phe-295, His-297 and Tyr-586. A direct inter-domain interaction is formed between His-297 and Tyr-586, while the interaction between Phe-295 and His-297 is necessary for the positioning of His-297 to interact with Tyr-586 (Supplementary Figure S4A). To investigate this, we purified *Mth-*Hel308^Y586A^ and assessed whether its activities are equivalent to Hel308^F295A^. Measuring DNA helicase activity in real-time using FRET (Figure 3C) confirmed that Hel308^F295A^ is a hyperactive helicase, in agreement with gel-based assays. Rates of DNA unwinding were 283 and 1090 pM (substrate) s^−1^ μM (helicase)^−1^ for wild type Hel308 and Hel308^F295A^, respectively. DNA unwinding by Hel308^Y586A^ was also hyperactive, measuring 760 pM (substrate) s^−1^ μM (helicase)^−1^, which corresponds to 2.7-fold hyperactivity over wild type Hel308. FRET measurements confirmed DNA annealing hyperactivity of Hel308^F295A^ (Figure 3D), observing an initial rate of 127 pM (substrate) s^−1^ μM (helicase)^−1^, compared to 36.0 pM (substrate) s^−1^ μM (helicase)^−1^) for wild type Hel308. However, Hel308^Y586A^ annealed DNA similarly to wild type Hel308, giving an initial rate of 40.2 pM (substrate) s^−1^ μM (helicase)^−1^. In EMSAs, Hel308^Y586A^ bound to the forked DNA similarly to wild type Hel308 (Figure 3E), which contrasts with hyperactive DNA binding observed from Hel308^F295A^ (Figure 2B and Supplementary Figure S4). Therefore, these two mutations in Hel308 can uncouple DNA helicase activity from DNA annealing, and indicate that Hel308 domain 2 motif IV controls DNA annealing.

### *Haloferax volcanii* cells lacking Hel308 are sensitive to mitomycin C and show increased homologous recombination

**Figure 4.**
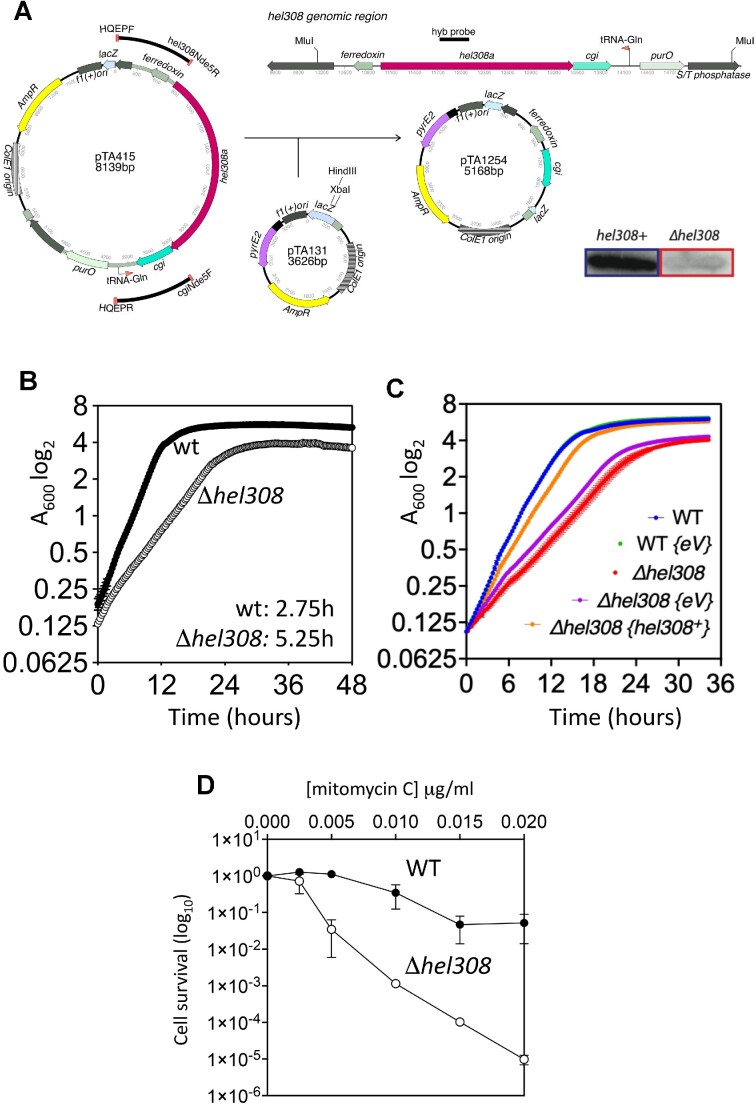
*Haloferax volcanii* cells lacking Hel308 are sensitive to mitomycin C and show increased homologous recombination. (**A**) The *H. volcanii hel308* genetic clone, pTA415, from which all *H. volcanii hel308* plasmids in this study are derived, comprises a 5.35 kb MluI chromosomal fragment containing *hel308a* and *cgi* cloned in pBluescript II SK+. Upstream and downstream PCR fragments were ligated together via the internal *Nde*I site and cloned in the *Hind*III and *Xba*I sites of pTA131 (26) to generate the *hel308* deletion plasmid, pTA1254. All plasmid constructs were verified by PCR. Deletion of *hel308* from *H. volcanii* was screened via colony hybridization using an internal *hel308* radio-labelled probe (ski2F/ski2R PCR product), 50% of 5-FOA-resistant colonies yielded the *Δhel308* allele, which was further verified via Southern blot (data not shown). (**B**) Cell deficient for *hel308* have slow growth compared to WT cells (H26 background). Generation time is indicated next to each strain. All strains (*n* = 2) were incubated on the same 96-well plate and growth measured simultaneously using a microplate spectrophotometer. (**C**) The growth defect of Δ*hel308* cells (red) is largely rescued by *in trans* expression of {plasmid-borne} *hel308^+^* (yellow, pTA2562). Inclusion in cells of the empty vector (purple, eV, pTA354) does not rescue this growth defect. All strains (*n* = 10) were incubated on the same 96-well plate and growth measured simultaneously. (**D**) *Δhel308* cells have increased sensitivity to MMC treatment compared to wild type cells (H26 background). Survival fraction is calculated relative to un-treated control. Each data point is generated as the mean of at least three independent trials. Standard deviation is shown.

**Figure 5.**
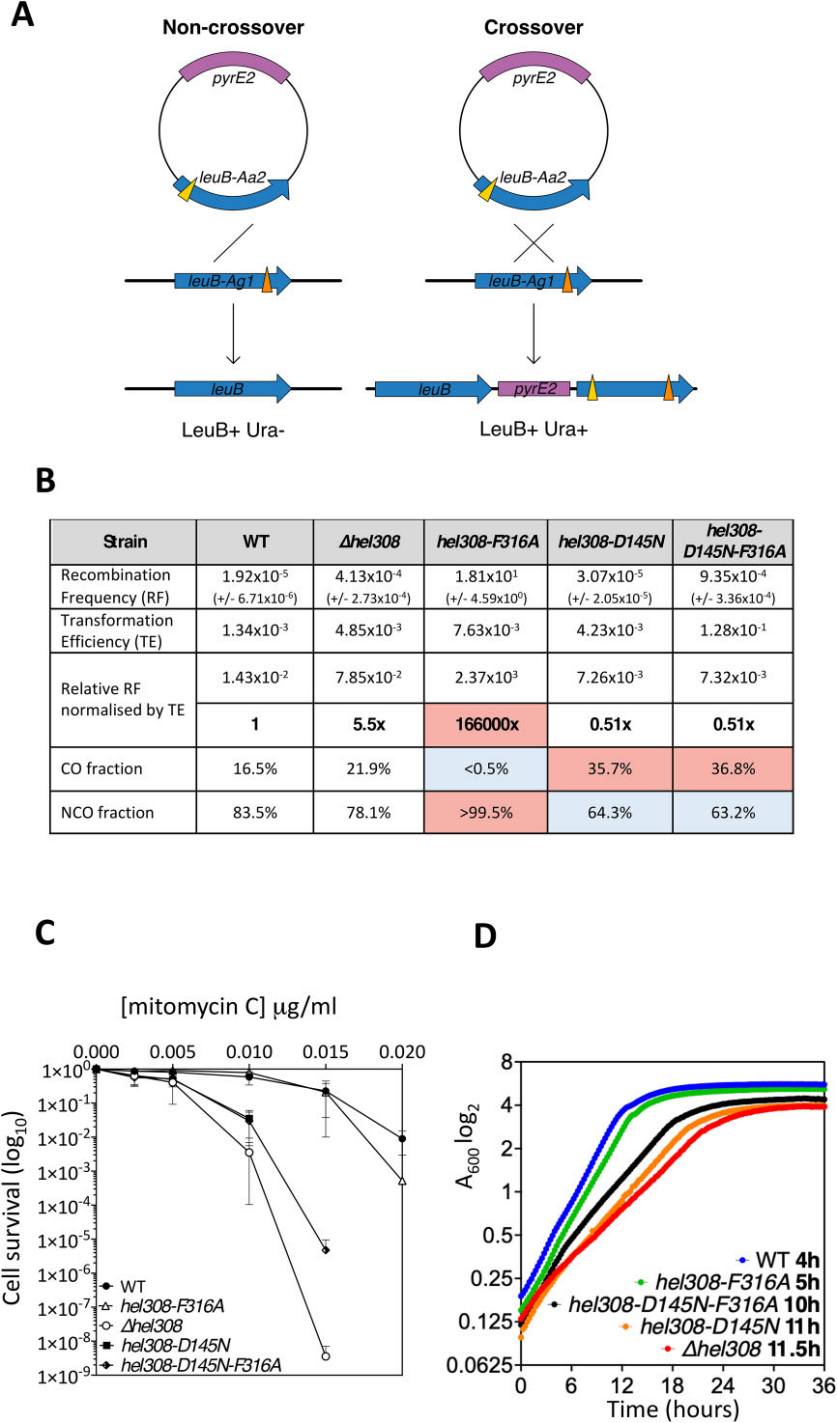
The separation-of-function mutation in motif IVa results in hyper-recombination as non-crossovers but has no effect on cell viability or sensitivity to DNA damage. (**A**) In the recombination assay, which is carried out in the H164 background, *ΔpyrE2* strains with a chromosomal *leuB-Ag1* allele (*leu-*) are transformed with pTA163, carries *pyrE2* and the *leuB-Aa2* allele (*leu-*). A recombination event between the plasmid and chromosomal mutant *leuB* alleles generates a wildtype *leu +* allele allowing strains to grow on media lacking leucine. The proportion of crossover (CO) and non-crossover (NCO) events are determined by the retention or loss of the *pyrE2* marker found on pTA163: CO are *pyrE2+ (ura+)*, and NCO are *ΔpyrE2 (ura-)*. (**B**) Values in bold measure recombination relative to wild type cells. *P* values were calculated using the t-test for recombination frequency (RF) and the chi squared test for CO:NCO fraction. Significant differences from the wildtype (*P*= 0.05) are blue for a decrease and red for an increase. Δ*hel308* cells demonstrate slightly increased recombination (*P =*0.25) with a similar CO:NCO fraction to wild type cells (*P =*0.18). The motif IVa mutant *hel308*^F316A^ dramatically increased recombination events (*P =*0.0043), which were observed only as NCOs (gene conversions) (*P =*0.0001). The ATPase/helicase inactive mutant *hel308*^D145N^ allele slightly decreased recombination (*P =*0.23) with an elevated CO ratio (*P =*0.0001). CO and NCO events are represented as a percentage of 200 colonies counted. Mean RF values are shown with the standard error in brackets (*n* = 5). (**C**) Walker B mutant *hel308* cells and *Δhel308* cells are similarly sensitive to MMC treatment (H164 background). The *hel308*^F316A^ allele alone does not affect cell survival after MMC treatment. Survival fraction is calculated relative to un-treated control. Each data point is generated as a mean of 3 independent trials. Standard deviation is shown. (**D**) Walker B mutant *hel308* cells are slow growing compared to wild type cells (H164 background). The *hel308*^F316A^ allele alone does not impede cell growth. Generation time is indicated next to each strain. All strains (*n* = 2) were incubated on the same 96-well plate and growth measured simultaneously.

We utilized these newly identified mutations in Hel308 to investigate the function of motif IV in living cells, turning to the extensive genetic tools available for the euryarchaeon *Haloferax volcanii* (HVO Hel308) (41). *H. volcanii* strains were generated where *hel308* was deleted (Δ*hel308*); care was taken to ensure that the overlapping essential gene *cgi* remained intact and that only *hel308* is deleted (Figure 4A). We observed that Δ*hel308* cells have a doubling time of 5.25 h, compared to 2.75 h for wild type cells (Figure 4B). This defect was rescued by complementation using Hel308 expressed *in trans* from a plasmid (Figure 4C). Consistent with Hel308 contributing to DNA repair during replication stress, Δ*hel308* cells were 10–100-fold more sensitive to killing by mitomycin C than wild type cells (Figure 4D). We next analysed whether Hel308 plays a role in homologous recombination, by generating *Δhel308* in a recombination-tester strain of *H. volcanii* (28). In this assay, recombination is measured between two different *leuB* (*leu-*) point-mutation alleles (Figure 5A). The chromosomal *leuB-Ag1* allele is carried by a strain that lacks *pyrE2* (*ura-*), while the *leuB-Aa2* allele is introduced via a plasmid that carries the functional *pyrE2* gene. Recombination between the plasmid and chromosomal *leuB* alleles generates a wildtype *leuB+* gene, allowing strains to grow on media lacking leucine; integration of the plasmid-borne *pyrE2* by crossing-over results in strains that are also able to grow on media lacking uracil. Plasmid × chromosome recombination in Δ*hel308* cells was found to be 5.5× more frequent than in wild type *hel308*^+^ cells (Figure 5B), suggesting that Hel308 antagonises homologous recombination in archaeal cells.

### Mutation of the Hel308 motif IV phenylalanine leads to dramatically increased gene conversion in *H*.*volcanii*

We next mutated *H. volcanii* chromosomal *hel308* to generate the *hel308*^F316A^ allele, which is equivalent to the *Mth-*Hel308^F295A^ described above. In addition, we generated a *hel308*^D145N^ allele, which lacks the catalytic Walker B/DEAD box required for ATPase and helicase activity. *H. volcanii hel308*^D145N^ mutant cells were found to be as sensitive to mitomycin C as Δ*hel308* cells, but *hel308*^F316A^ cells were no more sensitive to mitomycin C than the wild type, (Figure 5C). Therefore, Hel308 helicase activity is required for repair of DNA damage due to mitomycin C, but motif IVa does not play a role in DNA repair. However, plasmid x chromosome recombination in *hel308*^F316A^ cells was found to increase by 166000-fold (Figure 5B), which is significantly greater than the modest 5.5-fold increase observed in Δ*hel308* cells. To determine whether the dramatic increase in recombination in *hel308*^F316A^ cells was attributable to crossover or non-crossover (gene conversion) events, recombinants from the *leuB* heteroallele recombination assay were scored for chromosomal integration of the plasmid-borne *pyrE2* gene by crossover recombination (Figure 5A). We determined that the elevated recombination in *hel308*^F316A^ cells was due exclusively to non-crossover events, whereas wild type, *Δhel308*, and *hel308*^D145N^ mutant cells all showed both crossover and non-crossover events (Figure 5B). Therefore, motif IVa of Hel308 plays a role in the suppression of gene conversion, but not DNA repair, and we conclude that *hel308*^F316A^ is a separation-of-function mutation.

### The *hel308*^D145N^ allele is dominant to *hel308*^F316A^ in *H*.*volcanii*

Having identified separable DNA repair and recombination roles of Hel308, we investigated which function is dominant. Combining the helicase-inactive (*hel308*^D145N^) and hyperactive (*hel308*^F316A^) alleles to generate *hel308*^D145N/F316A^ resulted in a minor reduction in recombination that was indistinguishable from *hel308*^D145N^ (Figure 5B). Similarly, the *hel308*^D145N/F316A^ double mutant was hyper-sensitive to mitomycin C, mirroring the *hel308*^D145N^ allele rather than *hel308*^F316A^ (Figure 5C). Comparing growth rates (Figure 5D) showed similar generation times for wild type *hel308^+^* and *hel308*^F316A^ mutant cells (4 and 5h, respectively), while the generation time of the *hel308*^D145N/F316A^ double mutant was similar to *hel308*^D145N^ (10 and 11h, respectively). We conclude that Hel308 has defined and separable DNA damage repair and recombination functions, and that the ATPase-dependent DNA repair phenotype is dominant.

## DISCUSSION

**Figure 6.**
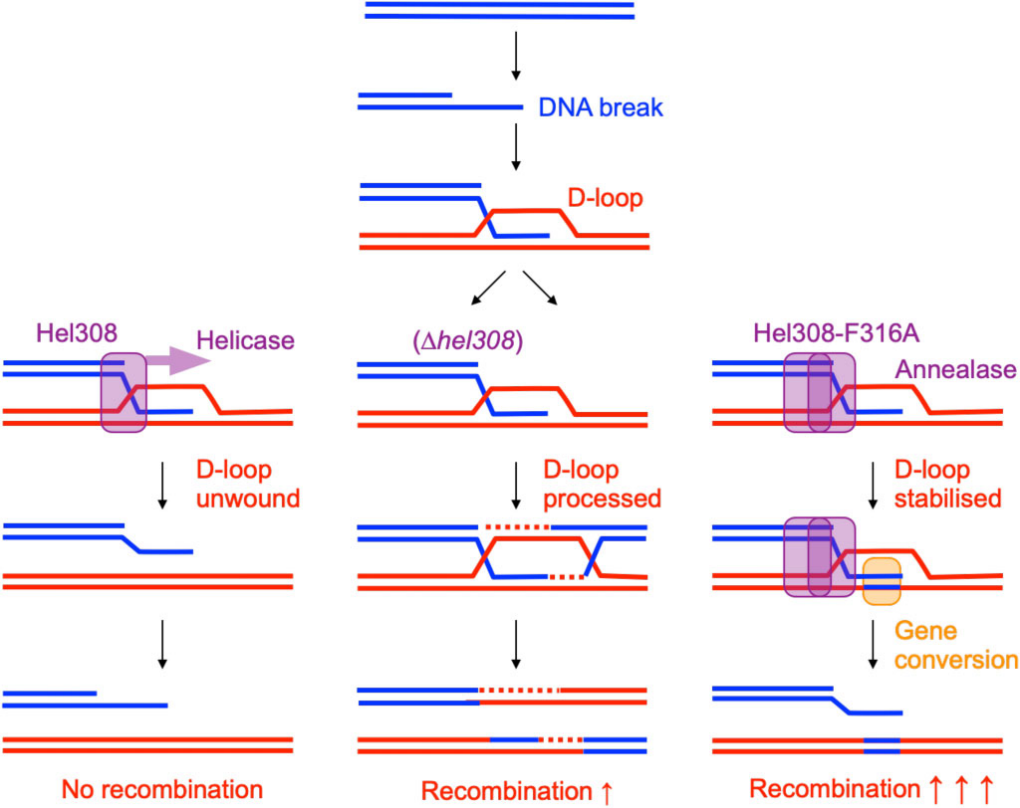
Separation-of-function mutations in motif IVa reveal that Hel308 has two distinct roles: repair of DNA damage, and suppression of HR. Proposed model, where wild type Hel308 actively targets and unwinds D-loops, antagonising recombination, and directing repair to an alternative pathway. In the absence of Hel308, D-loops are more readily processed by HR machinery, increasing the level of recombination modestly. The hyper-annealase Hel308^F316A^ stabilises strand exchange and DNA pairing in D-loops, facilitating gene conversion and thereby resulting in exceptionally high rates of (noncrossover) recombination.

We have shown here that in archaeal cells, the ATP-dependent DNA helicase Hel308 is required for the repair of mitomycin C induced DNA damage, and to control homologous recombination. These two functions of Hel308 in DNA repair and recombination are separable: the helicase-inactivating mutation Hel308^D145N^ resulted in sensitivity to mitomycin C but only a moderate decrease in recombination, whereas the motif IVa mutation Hel308^F316A^ increased recombination by 166000-fold whilst having no effect on mitomycin C sensitivity. Combining these mutations had the same effect as Hel308^D145N^ alone, indicating that the DNA repair function of Hel308 is dominant over the recombination function. This suggests that Hel308 helicase activity contributes to DNA repair by antagonising the formation of recombinogenic DNA products such as D-loops, consistent with both the ability of Hel308 to dissociate D-loop structures *in vitro* (9), and the modest increase in recombination we observed *in vivo* when *hel308* was deleted (Figure 6).

In addition to its activity as an ATP-dependent DNA helicase, Hel308 has the ability to anneal single stranded DNA independently of ATP. These activities of Hel308 mirror those of the human homologue HELQ, where the balance between helicase and annealase functions is controlled, at least in part, via interactions of HELQ with RPA and RAD51 (16). Our finding that a single amino change in motif IV (*Mth-*Hel308^F295A^, equivalent to *Hvo-*Hel308^F316A^) results in Hel308 that is hyperactive at DNA binding, annealing, and unwinding, reveals this motif IV as a critical controller of Hel308, specifically antagonising recombination and gene conversion. The hyper-recombination phenotype we observed in Hel308^F316A^ cells, and the requirement for helicase activity for DNA repair, suggest that the dramatic increase in recombination triggered by Hel308^F316A^ is caused by hyper-active DNA annealing. Such hyper-annealase activity could stabilise DNA pairing within D-loops or other homology-dependent DNA repair intermediates, prolonging the half-life of strand exchange and/or extending its range, and thereby facilitating gene conversion (Figure 6). This would account for the exceptionally high noncrossover recombination rate we observed in Hel308^F316A^ cells (Figure 5B). We speculate that this may also signpost how HELQ contributes to DNA repair and recombination in human cells, given high conservation of motif IV across Hel308 and HELQ proteins (Figure 1).

The widespread conservation of motif IV in several clades of Superfamily 2 helicases has been noted previously (42), and it has been proposed that this motif plays a role in maintaining the conformational rigidity of RecA2 domains. Our molecular dynamics simulations are consistent with this proposal, showing more open conformation in Hel308^F295A^, compared to wild type Hel308. Molecular dynamics simulations also highlighted interactions between motif IV Phe-295 (and the neighbouring motif IV histidine) with residues in the helicase ratchet domain 4. Such interactions would account for the hyperactive helicase activity of Hel308^F295A^, and its increased DNA binding affinity compared to wild type Hel308. Further biophysical analysis of this mutant protein will be required to establish the mechanism for hyper-annealing, which may result from conformational or oligomeric states that favour DNA annealing over ATP-dependent DNA unwinding, as was observed for the HELQ homologue (15). We conclude that archaeal cells utilize Hel308 to repair DNA damage *via* routes that avoid extensive recombination and gene conversion, and that motif IV controls the two activities of Hel308 as a helicase and annealase.

## Supplementary Material

gkad572_Supplemental_Files

## Data Availability

Homology modelled structures of *Mth-*Hel308 and *Mth-*Hel308^F295A^ after 1.0 μs of all-atom MD simulation have been deposited in the ModelArchive database with accession codes 10.5452/ma-w63zw (*Mth-*Hel308) and 10.5452/ma-rkamz (*Mth-*Hel308^F295A^). The template is the crystal structure of *A. fulgidus* Hel308 (PDB code 2P6R).
