# Supplementary material for: Archaeal Hel308 suppresses recombination through a catalytic switch that controls DNA annealing
Source: Nucleic Acids Res. 2023 Jul 6;51(16):8563–74. doi: 10.1093/nar/gkad572 (PMC10484726; doi:10.1093/nar/gkad572)

Supplementary Data

Archaeal Hel308 supresses recombination through a catalytic switch that controls DNA annealing

Rebecca J. Lever, Emily Simmons, Rebecca Gamble-Milner, Ryan J. Buckley, Catherine Harrison, Ashley Parkes, Laura Mitchell, Jacob A. Gausden, Sanja Škul, Branimir Bertoša, Edward L. Bolt and Thorsten Allers

Supplementary Figure Legends

Figure S1: Hel308 proteins and assays. (A). Purified Hel308 and Hel308 mutant proteins used in this work, as indicated. **(B).** Hyperactive helicase activity of Hel308^{F295A} is ATP dependent. The gel shows helicase products from 100 nM Hel308^{F295A} and 25 nM of forked DNA over time points from 0.5 to 10 minutes.

Figure S2. Molecular dynamics simulations comparing the flexibility of motif IVa amino acid residues in *Afu*-Hel308 wild type (WT) with Hel308^{F301A} (*Mth*-Hel308^{295A} equivalent, MUT). The experimentally determined atomic resolution structure of the wild type protein (PDB:2P6U) was utilized for the analyses. Further details of the modelling are in the main text results and methods. **(A).** Root mean square deviations (RMSD) of the backbone for the wild type *Afu*-Hel308 (WT, black lines) and Hel308^{F301A} (MUT, red lines) during the MD simulations. **(B-D).** Plots show Root Mean Square Fluctuation (RMSF) values for either the polypeptide backbone (labelled 'CA' shown in black and red for WT and MUT) or the amino acid residue sidechains (labelled 'HB2' shown in green and blue for WT and MUT); comparing motif IVa neighboring residues **(B).** Phe-301 and His-302, **(C).** His-302 with His-303, and **(D).** His-303 with Ala-304.

Figure S3: Molecular dynamics simulations using *Mth*-Hel308 sequences modelled from *Afu*-Hel308. (A and B). Plots are shown of the nanometer distances between amino acids in non-covalent interaction network linking domains 2 and 4: Thr237 (domain2)-Phe295 (domain2)-His297 (domain2) -Tyr567 (domain4), shown for: **(A)** wild type (WT) and **(B)** Hel308^{F295A} mutant. **(C).** Root mean square fluctuation (RMSF) analysis of MD simulations for *Mth*-Hel308 and *Mth*-Hel308^{F295A} either unbound to DNA or bound to DNA, as indicated, with the positioning of individual domains highlighted. The most significant differences in the wild type and mutant protein fluctuations are observed from the structures bound to DNA, depicted in red circles. **(D).** Root mean square deviations (RMSD) of the backbone for the wild type *Mth*-Hel308 and Hel308^{F295A} (RMSD) during the MD simulations, when using the structures either unbound or bound to DNA, as indicated. **(E).** Superposition of protein structures obtained after 1 μs of MD simulations of Hel308 wild type (domain 2 coloured dark blue and domain 4 coloured dark yellow) and Hel308 F295A mutant (domain 2 coloured light blue and domain 4 coloured light yellow). Amino acid residues Phe295 and His297 are shown as sticks and labelled. **(F).** Different orientation of protein structures obtained after 1 μs of MD simulations of Hel308 wild type (left) and Hel308 F295A mutant (right). Amino acid residues Phe295 and His297 are shown as sticks and labelled. Domain 2 (residues 194-403) is coloured blue and domain 4 (residues 498-634) is coloured yellow, the other domains are coloured grey.

Figure S4. EMSA showing that Hel308^{Y586A} binds to DNA similarly to wild type Hel308. This is in contrast with the hyperactive DNA binding of Hel308^{F295A}, repeated here in independent assay for direct comparison with Hel308^{Y586A} (see also Figure 2B). Proteins were each at 100, 200, 400 and 800 nM mixed with forked DNA (25 nM).

Supplementary Movie: *Archaeoglobus fulgidus* Hel308 bound to a partial DNA duplex. A movie of *A. fulgidus* Hel308 bound to a partial duplex DNA molecule (grey) was generated in PyMol using PDB accession code 2P6R. Hel308 domains are coloured as follows: RecA domains 1 and 2 (respectively, blue and green), winged helix domain 3 (red), helicase 'ratchet' domain 4 (orange), and domain 5 'brake' (salmon). Motif IVa residue R-groups are represented as yellow 'sticks' to highlight their proximity to one end of the alpha-helical domain 4 'ratchet' that is required for DNA translocation.

Supplementary Tables

Table S1: *H. volcanii* Strains

HVO Strain	Genotype	References/Source
H26	*ΔpyrE2*	(1)
H164	*ΔpyrE2, bgaHa-Bb, leuB-Ag1, ΔtrpA*	(2)
H438	*ΔpyrE2 {+pyrE2}*	This Study
H1391	*ΔpyrE2, Δhel308*	This Study
H2117	*ΔpyrE2, bgaHa-Bb, leuB-Ag1, ΔtrpA, Δhel308::trp+*	This Study
H2397	*ΔpyrE2, bgaHa-Bb, leuB-Ag1, ΔtrpA, hel308-F316A*	This Study
H2400	*ΔpyrE2, bgaHa-Bb, leuB-Ag1, ΔtrpA, hel308-D145N*	This Study
H3926	*ΔpyrE2, bgaHa-Bb, leuB-Ag1, ΔtrpA, hel308-D145N-F316A*	This Study
H5530	*ΔpyrE2, Δhel308, {+hel308, +pyrE2}*	This Study
H5533	*ΔpyrE2, Δhel308, {+pyrE2}*	This Study

Table S2: *E. coli* Strains

E. coli Strain	Genotype	Source
DH5α	*F– Φ80lacZΔM15 Δ(lacZYAargF) U169 recA1 endA1 hsdR17 (rK–, mK+) phoA supE44 thi-1 gyrA96 relA1 λ–*	Invitrogen
BL21-AI	*F– ompT hsdSB (rB-mB-) gal dcm araB::T7RNAP- tetA*	Invitrogen

Table S3: Plasmids

Plasmid	Use	References/ Source
pBluescript II SK+	Standard *E. coli* vector with functional blue/white screening capability	Stratagene
pET-14b	Standard *E. coli* vector for bacterial expression	Novagen
pEB431	MthHel308 protein expression vector	(3)
pEB465	MthHel308-F295A protein expression vector	This Study
pEB467	MthHel308-H267A protein expression vector	This Study
pEGS5	MthHel308-R591A protein expression vector	This Study

pTA2708	MthHel308-Y586A protein expression vector	This Study
pTA131	Integrative vector based on pBluescript II, with *pyrE2* marker	(1)
pTA163	Integrative vector containing *leuB-Ag2* and *pyrE2*. For use in recombination assays.	(2)
pTA354	Empty shuttle vector, control for episomal complementation assays	(4)
pTA415	pBluescript II SK+ with *H. volcanii* 5.35 kb MluI chromosomal fragment, containing *hel308* gene	This Study
pTA1254	*hel308* deletion construct, based on pTA131	This Study
pTA1273	*hel308::trpA+* deletion construct based on pTA1254	This Study
pTA1316	Subclone of 3.64 kb *hel308* chromosomal fragment from pTA415, cloned in pTA131	This Study
pTA1335	*hel308-D145N* gene replacement construct, based on pTA1316	This Study
pTA1642	*hel308-F316A* gene replacement construct, based on pTA1316	This Study
pTA1952	*hel308-D145N-F316A* gene replacement construct, based on pTA1316	This Study
pTA2562	Episomally expressed *hel308* in pTA354	This Study

Table S4: PCR Primers

Primer	Sequence	Purpose
HQEPF	GCCGAAGCTTTTGGGGCGCGTCGTCC	Forward PCR primer for upstream flanking region of *hel308a*, to generate *hel308a* deletion construct, contains HindIII site
HQEPR	CGGAATCTAGACGCAACGTTTACAAA TACCCGCG	Reverse PCR primer for downstream flanking region of *hel308a*, to generate *hel308a* deletion construct, containing XbaI site
hel308 Nde5R	CAGTTCGCATatgATCTCCCTTGG	Amplification of upstream region of hel308 introducing NdeI site
cgiNde5F	GTGATTTCatATGAGGCTCCTCG	Amplification of downstream region of hel308 introducing NdeI site
PBSF	GTAAAACGACGGCCAGT	Amplification of internal region of *hel308a*
HQ D145NR	TCGTTGGCCACGACGCAGGTGAGTTG	Generation of a *hel308-D145N* mutation
HQ D145NF	GTCGTGGCCAACGAGGTCCACCTC	Generation of a *hel308-D145N* mutation
ski2R	CGACCCATCATCTGGTGGACTTC	Amplification of internal region of *hel308a*
Hel308FInt	AGCGCTGGGAGGAGTACGGC	Amplification of internal region of *hel308a*

Hel308 F316AR	CGTGGTGGgcCGCCGCGCCTTTGGCGACC	Generation of a *hel308-F316A* mutation
Hel308 F316AF	CGCGGCGgcCCACCACGCGGGACTCGCCG	Generation of a *hel308-F316A* mutation
Hel308EcoR	AGGTAGTCGAGCACGCGGTCC	Amplification of internal region of *hel308a*
Ski2F	CCTCGCTCGTCTTCGTGAACTC	Amplification of internal region of *hel308a*

Table S5: DNA Substrates

**Oligonucleotide**	**Sequence 5' to 3'**	**Purpose**
MW12	Cy5-GTCGGATCCTCTAGACAGCTCCATGATCACTGGCA CTGGTAGAATTCGGC	Anneal to MW14 for helicase assays
MW14	CAACGTCATAGACGATTACATTGCTACATGGAGCTGTCT AGAGGATCCGA	Anneal to MW12 for helicase assays
ELB40	Cy5-GGAGCTCCCTAGGCAGGATCGTTCGCGACGATGG CCTTCGAAGAGCTCCAGTTACGGATACGGATCCTGC	DNA annealing assay with ELB71
ELB71	GCAGGATCCGTATCCGTAACTGGAGCTCTTCGAAGGCC ATCGTCGCGAACGATCCTGCCTAGGGAGCTCC-Cy3	DNA annealing assay with ELB70

Supplementary References

1. Allers, T., Ngo, H.P., Mevarech, M. and Lloyd, R.G. (2004) Development of additional selectable markers for the halophilic archaeon Haloferax volcanii based on the leuB and trpA genes. *Appl Environ Microbiol*, **70**, 943-953.
2. Lestini, R., Duan, Z. and Allers, T. (2010) The archaeal Xpf/Mus81/FANCM homolog Hef and the Holliday junction resolvase Hjc define alternative pathways that are essential for cell viability in Haloferax volcanii. *DNA Repair (Amst)*, **9**, 994-1002.
3. Guy, C.P. and Bolt, E.L. (2005) Archaeal Hel308 helicase targets replication forks in vivo and in vitro and unwinds lagging strands. *Nucleic Acids Res*, **33**, 3678-3690.
4. Norais, C., Hawkins, M., Hartman, A.L., Eisen, J.A., Myllykallio, H. and Allers, T. (2007) Genetic and physical mapping of DNA replication origins in Haloferax volcanii. *PLoS Genet*, **3**, e77.

S1

A

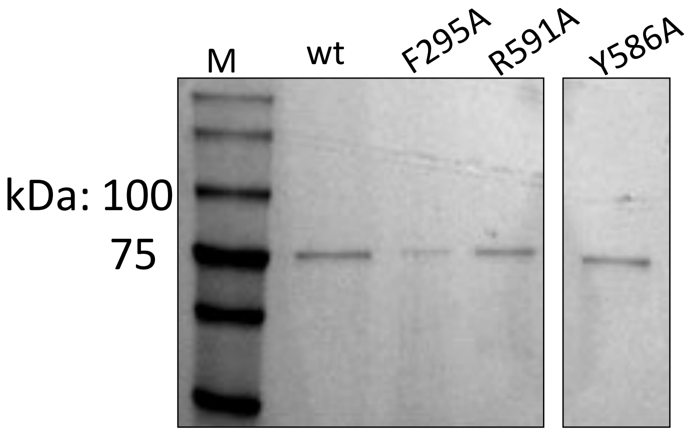

M
wt
F295A
R591A
Y586A
kDa: 100
75

B

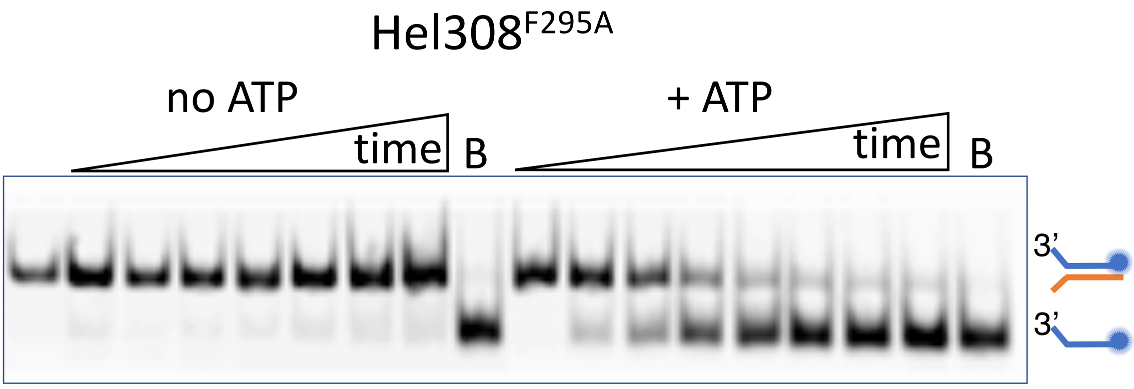

Hel308^{F295A}
no ATP
time
B
+ ATP
time
B
3'
3'

S2

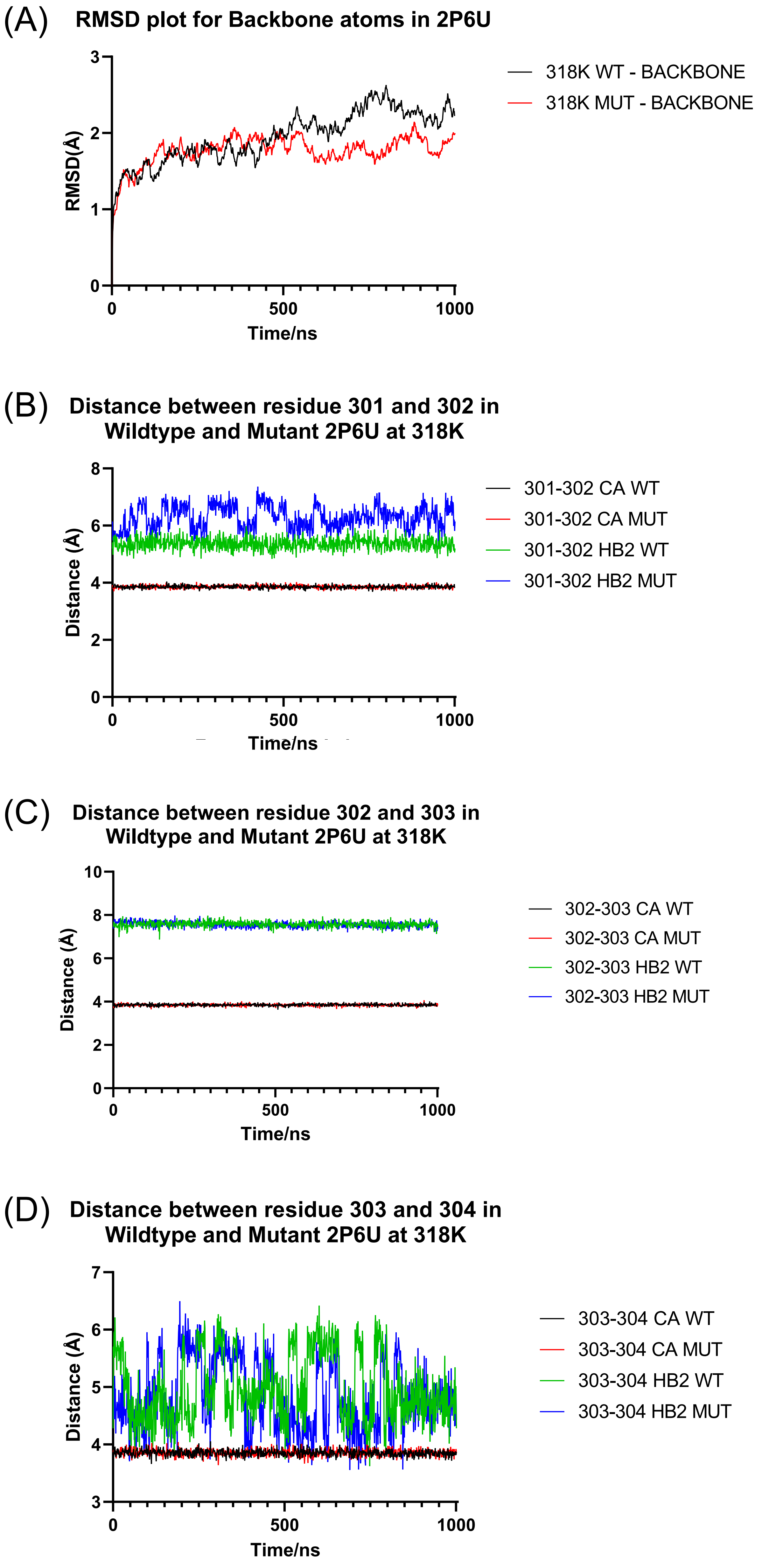
(A) RMSD plot for Backbone atoms in 2P6U
RMSD(Å)
Time/ns
318K WT - BACKBONE
318K MUT - BACKBONE
(B) Distance between residue 301 and 302 in Wildtype and Mutant 2P6U at 318K
Distance (Å)
Time/ns
301-302 CA WT
301-302 CA MUT
301-302 HB2 WT
301-302 HB2 MUT
(C) Distance between residue 302 and 303 in Wildtype and Mutant 2P6U at 318K
Distance (Å)
Time/ns
302-303 CA WT
302-303 CA MUT
302-303 HB2 WT
302-303 HB2 MUT
(D) Distance between residue 303 and 304 in Wildtype and Mutant 2P6U at 318K
Distance (Å)
Time/ns
303-304 CA WT
303-304 CA MUT
303-304 HB2 WT
303-304 HB2 MUT

S3

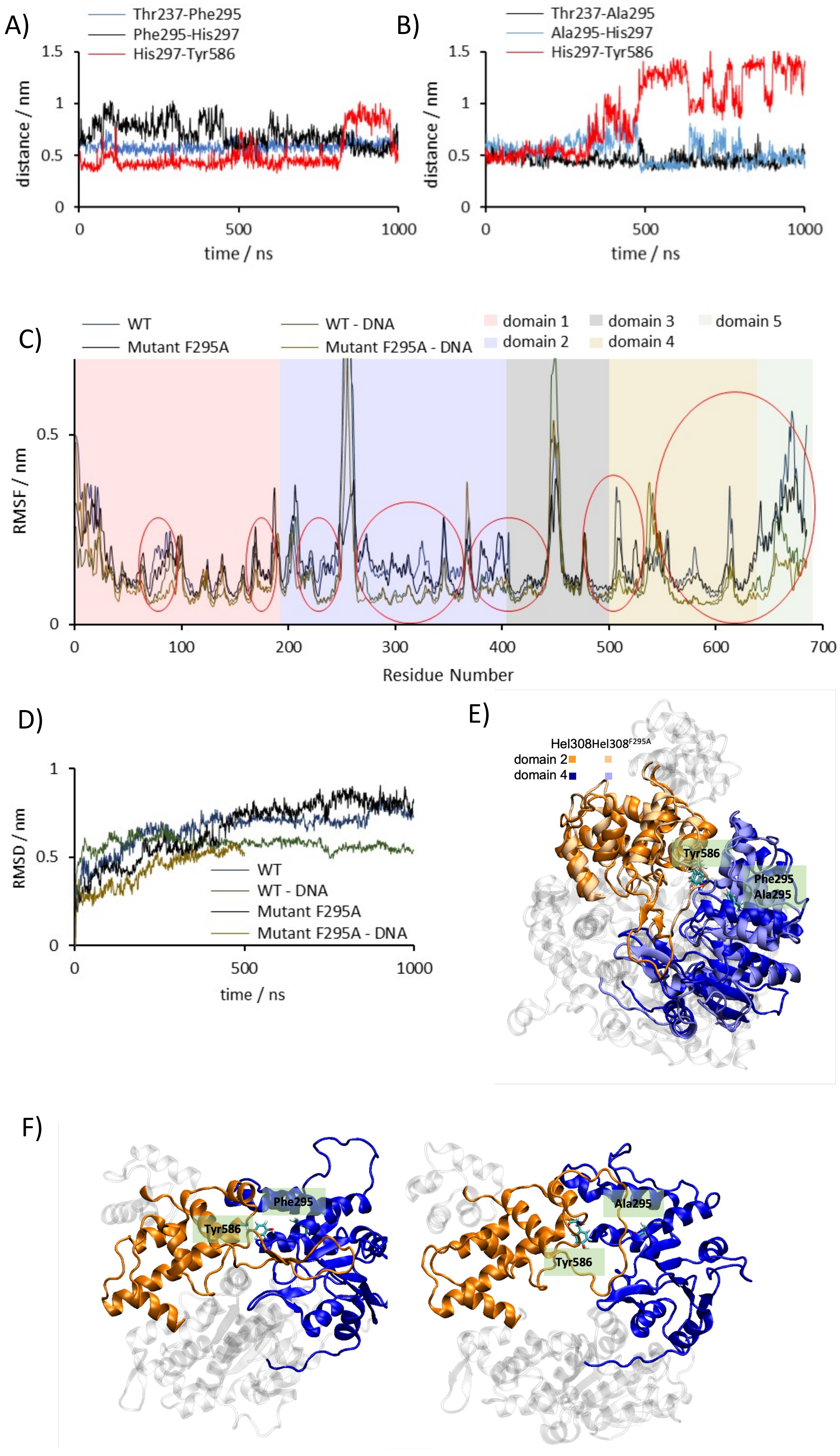

A)
Thr237-Phe295
Phe295-His297
His297-Tyr586
distance / nm
time / ns
B)
Thr237-Ala295
Ala295-His297
His297-Tyr586
distance / nm
time / ns
C)
WT
Mutant F295A
WT - DNA
Mutant F295A - DNA
domain 1
domain 2
domain 3
domain 4
domain 5
RMSF / nm
Residue Number
D)
RMSD / nm
WT
WT - DNA
Mutant F295A
Mutant F295A - DNA
time / ns
E)
Hel308 Hel308^{F295A}
domain 2
domain 4
Tyr586
Phe295
Ala295
F)
Phe295
Tyr586
Ala295
Tyr586

S4

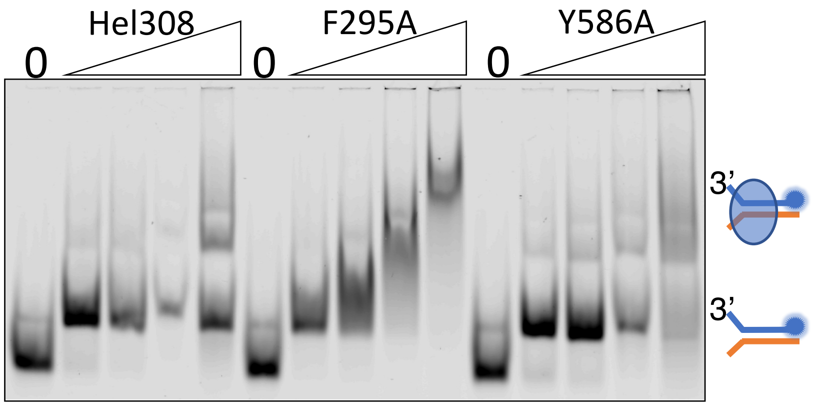

Supplement: gkad572_Supplemental_Files [file gkad572_supplemental_files.zip › NAR-00902-D-2023 Lever et al Suppl Data.pdf]
